# Femtomolar and locus-specific detection of N^6^-methyladenine in DNA by integrating double-hindered replication and nucleic acid-functionalized MB@Zr-MOF

**DOI:** 10.1186/s12951-021-01156-0

**Published:** 2021-12-07

**Authors:** Qingyuan Zheng, Tong Wang, Xinmin Li, Husun Qian, Xintong Bian, Xingrong Li, Huijie Bai, Shijia Ding, Yurong Yan

**Affiliations:** 1 Key Laboratory of Clinical Laboratory Diagnostics (Ministry of Education), College of Laboratory Medicine, Chongqing Medical University, Chongqing, 400016 China; 2 Department of Laboratory Medicine, Chongqing Hospital of Traditional Chinese Medicine, Chongqing, 400016 China

**Keywords:** N^6^-Methyladenine DNA, Double-hindered replication, MOF, Electrochemical biosensor

## Abstract

In this study, a novel electrochemical biosensor was constructed for ultrasensitive and locus-specific detection of N^6^-Methyladenine (m^6^A) in DNA using double-hindered replication and nucleic acid-coated methylene blue (MB)@Zr-MOF. Based on the combination of m^6^A-impeded replication and Ag^I^-mediated mismatch replication, this mode could effectively stop the extension of the strand once DNA polymerase encountered m^6^A site, which specifically distinguish the m^6^A site from natural A site in DNA. Also, Zr-MOF with high porosity and negative surface potential features was carefully chose to load cationic MB, resulting a stable and robust MB@Zr-MOF electrochemical tag. As a result, the developed biosensor exhibited a wide linear range from 1 fM to 1 nM with detection limit down to 0.89 fM. Profiting from the high sensitivity and selectivity, the biosensing strategy revealed good applicability, which had been demonstrated by quantitating m^6^A DNA at specific site in biological matrix. Thus, the biosensor provides a promising platform for locus-specific m^6^A DNA analysis.

**Supplementary Information:**

The online version contains supplementary material available at 10.1186/s12951-021-01156-0.

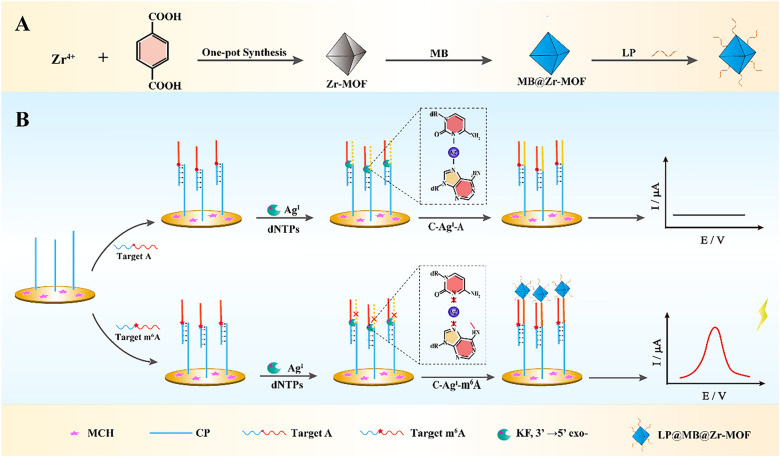

## Introduction

Epigenetic modifications on nucleic acids could significantly regulate the long-term gene activity and expression [1–3]. In particular, N^6^-methyladenine (m^6^A) DNA, methylated at the N^6^ position of adenine in DNA, is one of the most important and prevalent heritable epigenetic modifications in bacteria and higher eukaryotic cells [4, 5]. In human cells, m^6^A/A ratio is  ~ 0.056% in the blood genome DNA sample which means nM–pM levels of m^6^A DNA [6]. Recent studies have shown that m^6^A DNA is closely involved with DNA mismatch repair, chromosome replication, and cell-cycle regulation [7–9]. These characteristics enable m^6^A DNA to play an enigmatic role in physiological and pathological conditions, especially in the initiation and progression of cancers, such as ovarian cancer [10], liver cancer [11], and glioblastoma [12]. Thus, selective and sensitive quantitative analysis of m^6^A DNA is valuable for more understanding its biological functions and diagnosing cancers. However, it is still a challenge for the following reasons: the one is without any alteration in nucleotide sequence of m^6^A DNA, and the other is that m^6^A sites are not susceptible to chemical modifications [13, 14].

Previous methods for profiling of m^6^A DNA rely chiefly on next-generation sequencing [15–17] and modern mass spectrometry [18]. Although these strategies provided powerful tools to gain the distribution information of m^6^A sites in genome, the requirement of expensive instruments, professional technical staff, and long analysis time has impeded their wide applications. Aim at these issues, several methods have been developed. For instance, Yeh et al. introduced m^6^A-specific silver nanocluster beacon relying on the diverse stabilities between A/m^6^A–G base pairs [19]. Owing to the small difference of stability generated from methyladenine only, the capacity of distinction between m^6^A and A of the method was limit. Additionally, Zhou’ group reported primer extension-coupled electrophoresis separation strategy for the detection of m^6^A DNA utilizing the impediment of m^6^A to DNA synthesis [20]. Besides being unable to quantification and site-specific detection, this strategy was also difficult to distinguish m^6^A due to the fact that the optimal discernibility was gained in such a short extension time (1 min), which was attributed to the low selectivity of DNA polymerase towards m^6^A.

To improve the selectivity of m^6^A DNA detection, Ag^I^-mediated formation of adenine (A) and cytosine (C) mismatch (A–Ag^I^–C base pairs) by DNA polymerase has been applied. Specifically, DNA polymerase recognized Ag^I^-mediated base pairs resulting in the incorporation of a mismatched base into DNA primers through replication [21, 22]. In contrast, m^6^A site interfered the formation of the mismatch because of the steric hindrance caused by the bulky methyl group, which disturbed the recognition of DNA polymerase and stopped the replication [14]. Expanding the principle to ligase, Ag^I^-mediated ligation and rolling circle amplification (RCA) [14] and Ag^I^-assisted ligation coupled with hybridization chain reaction (HCR) [23] have been reported. Despite the significant difference between m^6^A and A in Ag^I^-mediated ligation, the high nonspecific reactions of RCA and HCR need to be avoided [24, 25]. Also, the sensitivities of these strategies are insufficient owing to the ultralow m^6^A DNA amounts [4].

Metal–organic frameworks (MOFs) are a class of new organic/inorganic porous materials with high porosity, large surface area, and multiple functionalities [26–28]. Particularly, Zr-MOF with negative surface potential is an ideal carrier for adsorbing massive amounts of cationic tags such as methylene blue (MB), which is capable of enhancing its analysis sensibility [29–31]. On the other hand, thanks to the existence of Zr-P bond, Zr-MOF easily links to DNA strands [32]. Considering these unique characteristics, Zr-MOF is regarded as a promising material for constructing high-performance nucleic acid biosensors [33, 34]. Although MOF-based strategies have been developed for the detection of m^6^A DNA [35, 36], these methods still face the problems: low sensitivity, high cost owing to the usage of antibody, and the failure of sensing specific m^6^A sites. Thus, exploring MB@Zr-MOF as electrochemical tag provides a new way for developing m^6^A DNA detection method.

Inspired by these, we developed a novel electrochemical biosensor for ultrasensitive and site-specific detection of m^6^A DNA based on double-hindered replication and nucleic acid-functionalized MB@Zr-MOF. In this sensing system, once Klenow fragment (KF) DNA polymerase encountered m^6^A sites, the extension of capture probes (CP) hybridized with the part sequence of m^6^A DNA was terminated due to the double-hindered replication (the impediment of m^6^A to DNA synthesis and the failed recognition by DNA polymerase resulting from the instability of m^6^A–Ag^I^–C mismatch in the absence of dTTPs). As a result, the remaining sequence of the m^6^A DNA was accessible. Then, linker probes (LP)-coated MB@Zr-MOF (LP@MB@Zr-MOF) bound to the free part of m^6^A DNA, thus gaining amplified electrochemical signal. Benefiting from the effective double-hindered replication and powerful signal amplification ability of MB@Zr-MOF, the developed biosensor provides a robust platform for femtomolar, single-base level and highly selective detection of m^6^A DNA without the involvement of any antibodies. Moreover, the biosensor holds the ability of site-specific m^6^A DNA detection by simply adjust the sequence of capture probe.

## Experimental section

### Material and reagents

Klenow Fragment DNA polymerase (3′ → 5′ exo^−^), 10 ×  Klenow buffer (500 mM Tris–HCl, 50 mM MgCl_2_ and 10 mM DTT, pH 7.9), and 10 ×  CutSmart™ buffer (20 mM Tris–acetate, 500 mM potassium acetate, 10 mM magnesium acetate and 100 µg/mL BSA, pH 7.9) were obtained from New England Biolabs (Beijing, China). GoldView I, 20 bp DNA marker, and dATPs, dTTPs, dCTPs and dGTPs were purchased from SBS Genetech Co., Ltd., (Beijing, China), TaKaRa (Dalian, China), and Thermo Fisher Scientific Inc., (Waltham Mass, USA), respectively. Mercapto hexanol (MCH) and MB were provided by Sangon Biotech Inc. (Shanghai, China). All oligonucleotides (HPLC grade) used in this experiment were synthesized by Sangon Biotech Inc. (Shanghai, China) and TaKaRa (Dalian, China). The DNA sequences were illustrated in Additional file 1: Table S1. Milli-Q water (resistance of  > 18 MΩ cm) was used to prepare all solutions. All cell lines including normal human stem cells (NeHepLxHT) and liver cancer cells (HepG2) were obtained from American Type Culture Collection (ATCC, USA). The MethylFlash m^6^A DNA Methylation ELISA Kit (Colorimetric) was purchased from Epigentek (NY, USA).

### Apparatus

Oligonucleotide concentrations were evaluated by a NanoDrop 1000 spectrophotometer (Thermo Scientific Inc., Wilmington, DE, USA). The electrophoretic gels were imaged by a ChemiDoc XRS system (Bio-Rad, Hercules, CA, USA). All electrochemical measurements were performed on a CHI660D Electrochemical Workstation (Shanghai Chenhua Instrument Co., Ltd., China) with a three-electrode electrochemical system including saturated calomel electrode as reference electrode, platinum electrode as counter electrode, and gold electrode (GE, 3 mm in diameter) as working electrode.

### Synthesis of LP@MB@Zr-MOF

First, Uio-66 was synthesized according to the previous report with the slight modification[18]. A mixture of 75 mg zirconium tetrachloride (ZrCl_4_), 782 mg benzoic acid, and 58 mg 2-aminoterephthalic acid (NH_2_-BDC) was added into N, N′-dimethylformamide (DMF, 9 mL) in a 20 mL Teflon-lined stainless-steel reactor. After sonicated the mixture for about 10 min, the reactor was heated at 80 °C for 12 h, and held at 100 °C for another 24 h. After cooling down to room temperature, the resultant solid was collected by centrifugation for 5 min at 8000 rpm, then washed thoroughly with DMF and methanol. The gained Zr-MOF was placed under vacuum at 80 °C to remove the volatile methanol. Afterwards, MB@Zr-MOF was prepared by mixing 0.15 g Zr-MOF and 0.06 g MB in 50 mL methanol at 80 °C for 48 h. The solid was then filtered off, extensively washed with deionized water and methanol, and finally vacuum at 60 °C overnight. To synthesize LP@MB@Zr-MOF, the prepared MB@Zr-MOF was dissolved in 1 × PBS solution (pH 7.9) to form a 1 mg/mL of MB@Zr-MOF solution, then 1 μM of LP was added in the solution and the mixture was stirred at 4 °C overnight. Subsequently, the baby blue product was collected by centrifugation (5 min, 8000 rpm) and then washed with 1 ×  PBS solution. Finally, the synthesized LP@MB@Zr-MOF was redissolved in 1 ×  PBS solution and stored at 4 °C for further use.

### Fabrication of the proposed electrochemical biosensor

First, GE was polished with 0.05 μM alumina powder for 5 min to a mirror, followed by sonication in ultrapure water, absolute ethanol and deionized water for 5 min, respectively. GE was then soaked in piranha solution for 10 min and rinsed thoroughly with deionized water for eliminating other impurity. 0.5 μM CP was pretreated by Tris (2-carboxyethyl) phosphine (50 μM) for 1 h to break disulfide bond. Subsequently, 10 μL of 0.5 μM CP was dropped on the cleaned electrode followed by the incubation at 4 °C overnight. The prepared electrodes were washed with 1 ×  PBS solution (pH 7.4) and then covered with 10 μL of 1 mM MCH for 0.5 h in room temperature to block the unbinding sites followed by rinsing with washing buffer.

For the m^6^A DNA detection, 10 μL of different concentrations of target was dropped onto the surface of the modified electrodes and incubated for 1 h at 37 °C to capture the target. Then, 10 µL of reaction buffer including AgNO_3_ (100 μM), dNTP (1 μM), KF (5 U), 10 mM Magnesium acetate, 8 mM DTT, 10 mM Tris-AcOH, and 100 mM AcOHNa, PH  = 7.9) was added to the electrode surface and incubated at 37 °C for another 15 min for performing the Ag^I^-mediated DNA mismatch and replication. Subsequently, the electrode was incubated in 1 mg/mL of LP@MB@Zr-MOF solution at 37 °C for 1 h. Eventually, the electrodes were washed with 1 ×  PBS solution to remove unbound LP@MB@Zr-MOF for subsequent measurement.

### Electrochemical measurements

Cyclic voltammetry (CV) and electrochemical impedance spectroscopy (EIS) measurements were conducted in 0.5 M KNO_3_ solution containing 0.5 mM [Fe (CN)_6_]^3/4−^. CV curves were recorded at scan rate of 10 mV s^−1^ and EIS spectra were collected with the frequency range from 0.1 MHz to 0.01 Hz. Meanwhile, the differential pulse voltammetry (DPV) measurements were performed in the working solution (0.1 M PBS, pH 7.4) with a pulse period of 0.5 s, a pulse width of 0.2 s, a pulse amplitude of 50 mV, and potential scan from − 0.6 to 0.2 V.

### Gel electrophoresis

Twelve percent native polyacrylamide gel (PAGE) was prepared to verify the extension of primer by KF DNA polymerase and the effective distinguishment between target DNAs and target m^6^A DNAs. Electrophoresis was carried in 1 ×  TBE buffer (2 mM EDTA, 89 mM Tris-boric acid, pH 8.3) at a 100 V constant voltage for 45 min. Then, the gel was immersed in a freshly prepared stain solution (80 mL of 1 ×  TBE buffer containing 4 μL of GoldView I) for 30 min. Afterwards, the gel was imaged using gel image system.

### Cell culture and DNA extraction

The selected cells were cultured in DMEM containing 10% FBS and supplemented with 100 μg/mL streptomycin and 100 U/mL penicillin at 37 ℃ in a humidified incubator containing 5% CO_2_. The extraction steps of DNA from cells as follows: 1 × 10^6^ HepG2 cells or NeHepLxHT cells was rinsed using cold PBS, and collected by centrifugation. After washing twice, 5 ml of DNA extraction buffer (10 mM Tris–HCl, 0.1 M EDTA, 0.5% SDS) were added. Then, 25 μL of proteinase K (100 μg/ml) was added and incubated for 3 h at 50 ℃. After that, a mixture of phenol, chloroform and isopentyl alcohol was used to extract the aqueous phase by centrifugation at 2500 r/min. Equal volume of LiCl (5 M) was added and incubated with ice bath for 10 min. The mixture was centrifuged at 2500 r/min for 10 min and the supernatant was transferred to a centrifuge tube which contained equal volume of isopropanol. After incubating for 10 min at room temperature, the product was collected through centrifugation at 2500 r/min for 10 min. Then, 0.1 times the volume of sodium acetate (3 M, pH  = 5.2) and 2 times the volume of – 20 ℃ precooling anhydrous ethanol were added and the mixture was incubated at – 20 ℃ for 20 min. At last, the DNA extraction was separated by centrifugation at 12,000 r/min for 5 min and was redissolved in 1 ml TE buffer for further use.

### Detection of m^6^A DNA in cells

Different concentrations of cell’s DNA extraction were tested using the proposed electrochemical biosensor and ELISA kit. The detection steps of the biosensor were according to the procedure proposed in “Fabrication of the proposed electrochemical biosensor” Section. ELISA analysis was conducted based on the product’s principle and procedure.

## Results and discussion

### Operation principle of the electrochemical biosensor

**Scheme 1:**
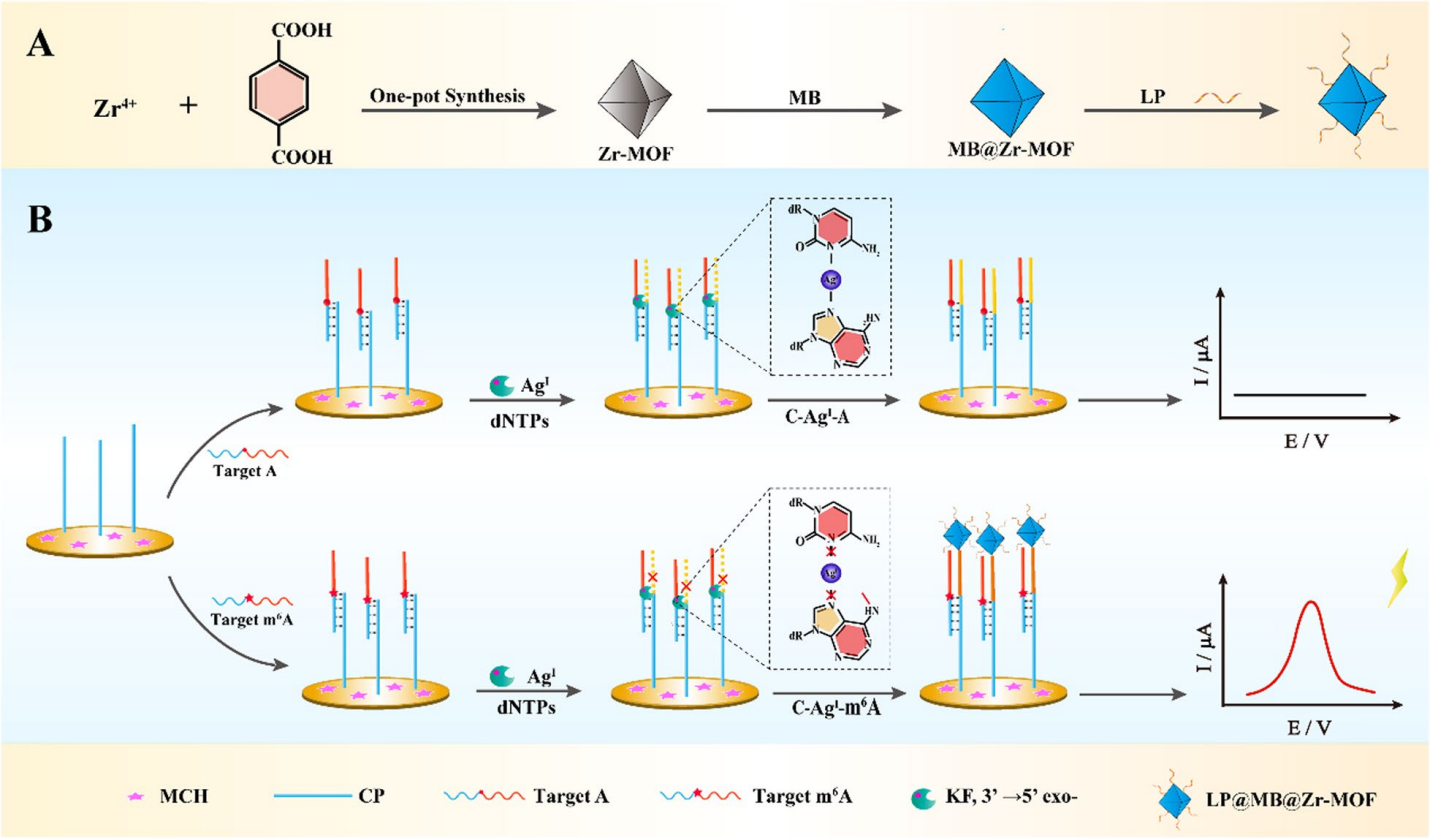
**A** The preparation of LP@MB@Zr-MOF electrochemical tag. **B** Schematic illustration of the biosensing protocol for m^6^A DNA detection

The principle of the electrochemical biosensor for ultrasensitive m^6^A DNA detection at a specific site by combining double-hindered replication with LP@MB@Zr-MOF was illustrated in Scheme 1. In part A, Zr-MOF was synthesized by using a facile one-pot solvothermal reaction with NH_2_-BDC as the ligand and Zr^4+^ as the metal nodes. Then, massive amounts of electroactive substrate MB were packed into Zr-MOF owing to its negative surface potential and high porosity, forming MB@Zr-MOF complex. Subsequently, the LP was linked with MB@Zr-MOF through Zr–P bonds, forming LP@MB@Zr-MOF electrochemical tag. The biosensing steps were shown in part B. Target DNA which was divided into two parts including CP and LP recognition domain by m^6^A site or A site was introduced and hybridized with CP. When there was an A site on the target DNA, KF DNA polymerase stabilized and replicated the mismatch A–C with the existence of enough Ag^I^ and dNTPs (without dTTP). As a result, the LP recognition domain on target DNA would be blocked, leading to the failed binding of LP@MB@Uio-66 to electrode surface. However, when there was a m^6^A site on target DNA, the incorporation of the mismatched C base into CP was stopped owing to the double-hindered replication caused by m^6^A and the instability of m^6^A–Ag^I^–C. Therefore, LP recognition domain hybridized with the LP@MB@Zr-MOF, producing obvious electrochemical signal. Through the sensing strategy, target DNA with specific m^6^A site was clearly distinguished from these with normal A site. Integration of the unique double-hindered replication and the excellent electrochemical activity of MB@Zr-MOF, the strategy realized ultrasensitive detection towards specific m^6^A site in DNA.

### Characterization of the LP@MB@Zr-MOF

**Fig. 1:**
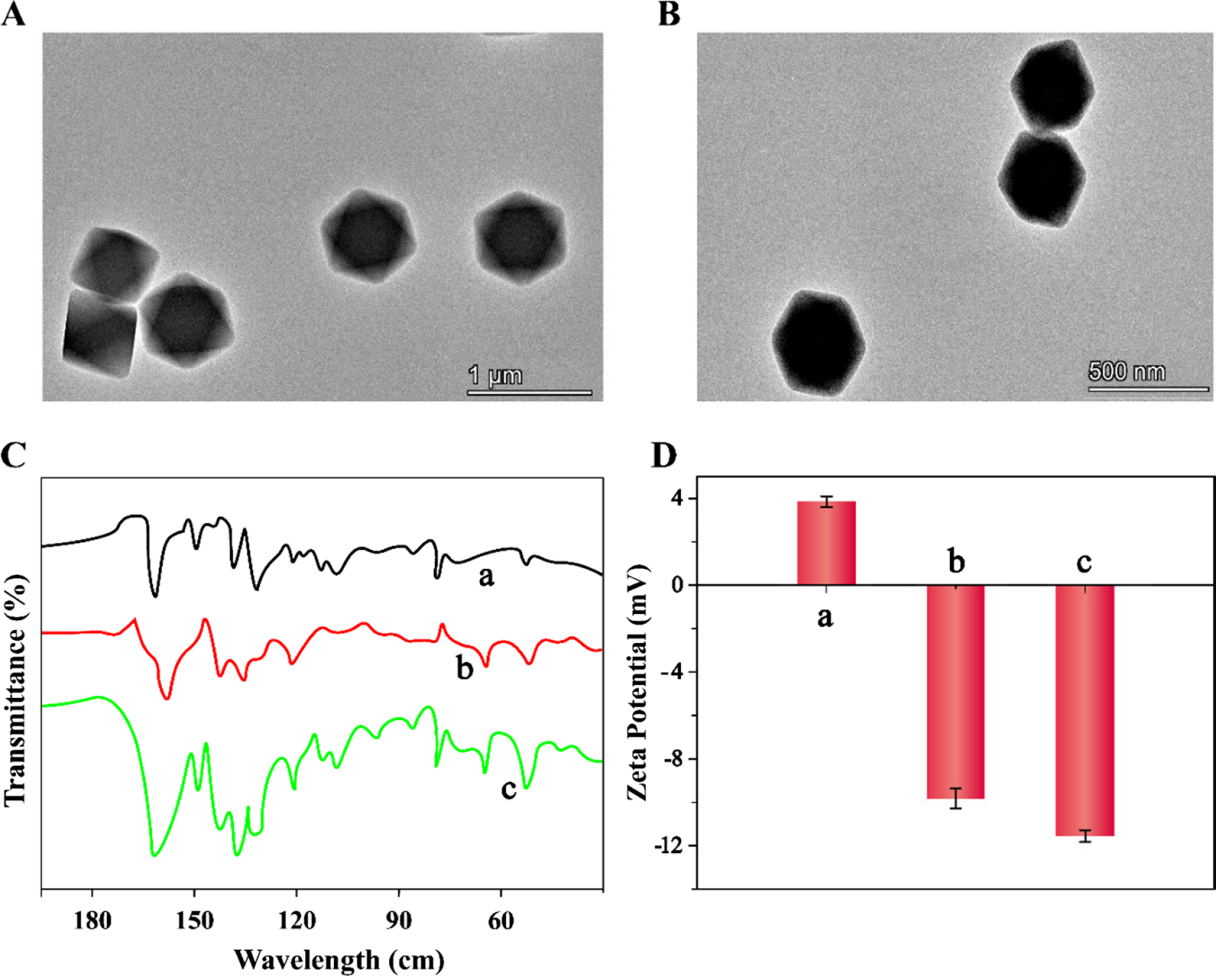
Characterization of the LP@MB@Zr-MOF. **A** TEM image of the Zr-MOF and **B** MB@Zr-MOF. **C** FT-IR spectra results of **a** MB, **b** Zr-MOF and **c** MB@Zr-MOF. **D** Zeta characterization values of **a** Zr-MOF, **b** LP@Zr-MOF and **c** LP@MB@Zr-MOF

Transmission electron microscopy (TEM), FT-IR spectra, and Zeta potential experiment were performed to characterize the LP@MB@Zr-MOF. TEM images showed that the Zr-MOF (Fig. 1A) and MB@Zr-MOF crystals (Fig. 1B) had identical diameter, demonstrating that the little influence of MB on the morphologies of the prepared Zr-MOF. As depicted in Fig. 1C, FT-IR spectra exhibited the new peaks in the MB@Zr-MOF compared with that in Zr-MOF, indicating that MB was effectively encapsulated in Zr-MOF. As illustrate in Fig. 1D, the Zeta potential value of the Zr-MOF, LP@Zr-MOF, and LP@MB@Zr-MOF was about 3.8 mV, − 10.32 mV, and − 11.82 mV, respectively, demonstrating that Zr-MOF was modified by LP and MB. These results verified the successful synthesis of the LP@MB@Zr-MOF electrochemical indicator.

### Characterization of the modification procedure on an electrode

CV and EIS were performed to characterize the stepwise electrode modification. Additional file 1: Fig. S1A gave the CV curves of various electrodes. It was obvious that a pair of redox peaks were observed at  ~ 0.14 V and  ~ 0.24 V on the GE (curve a). When the CP and MCH were immobilized on the electrode surface by turn, the peak current continued to decrease (curve b and c). Then, the binding of m^6^A DNA to the modified electrode leaded to a decrease of peak current again (curve d). It might be explained that biomolecular modification blocked the electron-transfer between the electrolyte and electrode. However, when the LP@MB@Zr-MOF electrochemical indicator hybridized with the m^6^A DNA, the peak current increased (curve e) owing to the electroactivity of MB@Zr-MOF. To evaluate the influence of biomolecules anchored on the electrodes, EIS was carried out (Additional file 1: Fig. S1B). The diameter of semicircle represented the electron-transfer resistance (Ret). It was found that the biomolecules on the electrode surface restricted the charge transfer when they were immobilized on the electrode surface. However, when the LP@MB@Zr-MOF was introduced, the Ret decreased (curve e). These results not only demonstrated the success of modification processes, but preliminarily confirmed the feasibility of the developed biosensor.

### Feasibility of the sensing strategy for m^6^A DNA detection

**Fig. 2:**
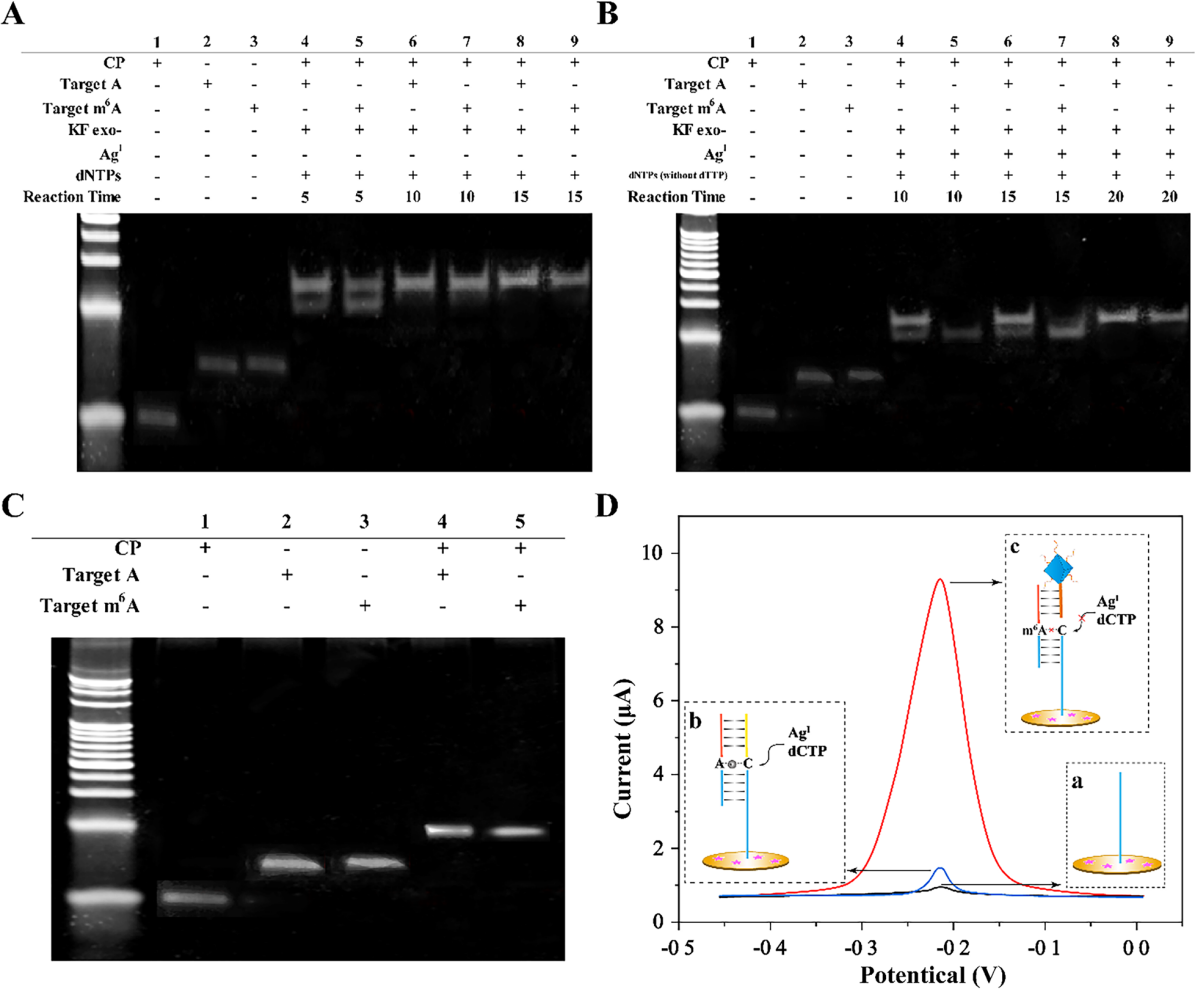
Investigation of the feasibility of the developed biosensor. **A**–**C** Verification of the high selectivity of double-hindered replication towards target m^6^A DNA by PAGE experiments. The concentrations of all DNA substrates were 1 μM except target. **D** DPV signals responding to **a** blank control, **b** 1 nM target A, and **c** 1 nM target m^6^A DNA

First, the double-hindered replication was investigated by PAGE experiment. As shown in Fig. 2A–C, lane 1, 2 and 3, in which each had a single band, represented CP, target A (wild-type DNA), and target m^6^A DNA, respectively. See the results in Fig. 2C, the band location of the dsDNA-1 hybridized by CP and target A (lane 4) is consistent with that of dsDNA-2 constructed by CP and target m^6^A DNA (lane 5), indicating that m^6^A site did not affect the hybridization of dsDNA. As seen in Fig. 2A, dsDNA, KF exo^−^, and dNTPs were mixed and incubated at 37 °C for 5 min (lane 4 and 5), 10 min (lane 6 and 7) and 15 (lane 8 and 9) min. However, both dsDNA-1 and dsDNA-2 were replicated in the first 5 min extension reaction. Therefore, target m^6^A DNA could not effectively be distinguished only depending on the impediment of m^6^A to DNA synthesis. As shown in Fig. 2B, when KF exo^−^, Ag^I^, dNTPs (without dTTP), and dsDNA were mixed at 37 °C for 10 min (lane 4 and 5), 15 min (lane 6 and 7) and 20 min (lane 8 and 9). Lane 4–7 indicated that dsDNA-1 was extended, but the extension of dsDNA-2 was hindered. Thus, target m^6^A DNA was distinguished effectively based on the double-hindered replication system.

Second, each reaction process of the sensing strategy was analyzed by measuring DPV signal, and the results were depicted in Fig. 2D. Compared to blank control that did not include target A and target m^6^A DNA (curve a), a very low DPV signal was detected responding to 1 nM target A (curve b), which was because the Ag^I^-mediated replication blocked LP recognition domain. However, in the presence of 1 nM target m^6^A DNA, a significant DPV signal was observed (curve c). This was attributed to the binding of LP@MB@Zr-MOF to LP recognition domain, generating amplified electrochemical signal. These results clearly demonstrated that the constructed biosensor possessed outstanding ability to specifically distinguish m^6^A DNA.

### Optimization of the experiment variables of the biosensing strategy

**Fig. 3:**
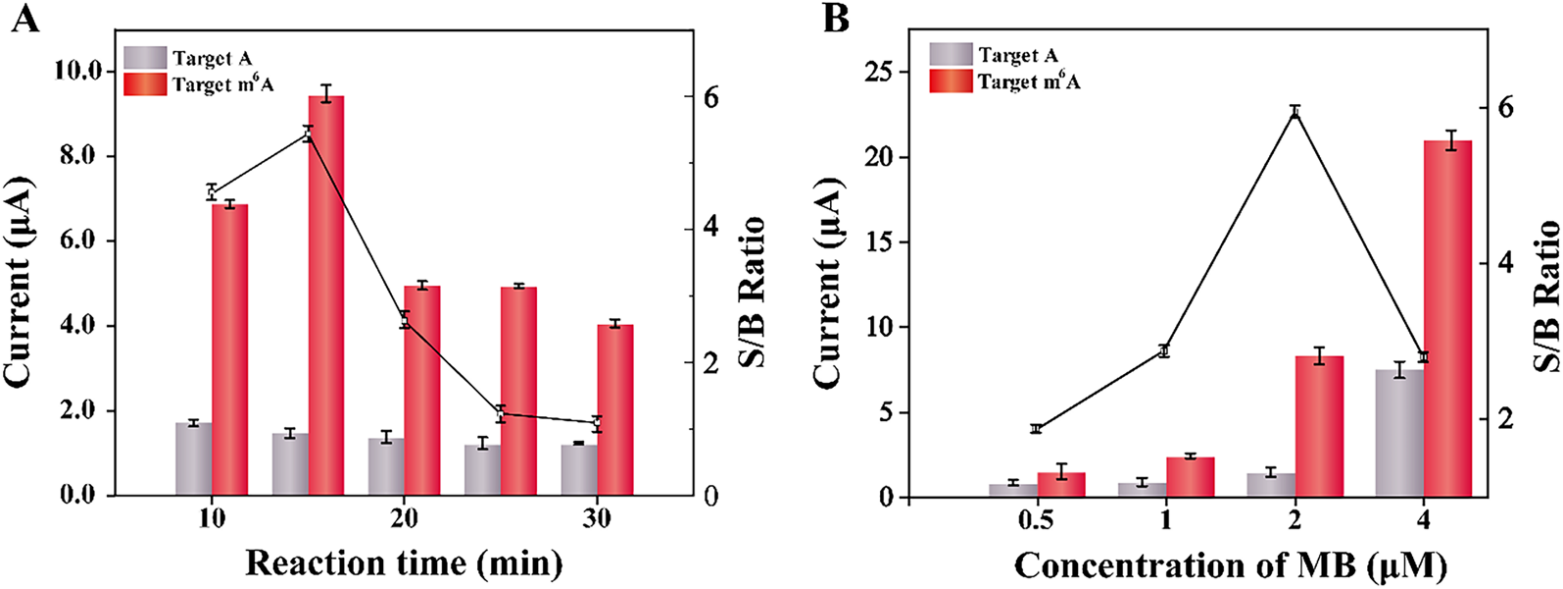
Optimization of the experimental parameters. Effect of **A** the double-hindered replication time and **B** concentrations of MB loaded on Zr-MOF on DPV signal responding to 1 nM target m^6^A DNA. All results expressed as mean  ±  standard variation (n  = 3)

To obtain the best performance of the electrochemical biosensor, three important experimental parameters were optimized. First, the double-hindered replication time was evaluated. As illustrated in Fig. 3A, the DPV signal increased with the incubation time until 15 min when the signal from target m^6^A DNA to background from target A (S/B) ratio reached the pinnacle. After that, the DPV signal decreased, because the DNA polymerase nonspecifically prolonged the CP if the incubation time was too long.

Second, the concentration of MB loaded on Zr-MOF was optimized. As shown in Fig. 3B, DPV signal exhibited an upward trend with the increase of MB concentration from 0.5 to 4 μM MB. However, when the concentration of MB surpassed 2 μM, an obvious background noise was observed, resulting from the nonspecific adsorption of superfluous MB on the electrode surface. Thus, the biggest S/B ratio was obtained when the concentration of MB loaded on Zr-MOF was 2 μM.

Third, we evaluated the influence of Zr-MOF concentration on DPV signal. As depicted in Additional file 1: Fig. S2, the different concentrations of Zr-MOF from 0.5 to 4 mg/mL were selected to screen the best concentration with optimal S/B ratio. Clearly, 1 mg/mL of Zr-MOF generated the most satisfactory S/B ratio that was taken as the best condition for the subsequent experiment. Next, the reaction buffer containing various concentration of AgNO_3_ from 50 to 150 μM was tested. As shown in Additional file 1: Fig. S3, the DPV signal and the S/B ratio increased gradually with the concentration of AgNO_3_ and reached the highest peak at 100 μM AgNO_3_. After that, the signal declined sharply. Thus, the reaction buffer containing 100 μM AgNO_3_ was selected for other experiments.

### Sensitivity of the developed biosensor

**Fig. 4:**
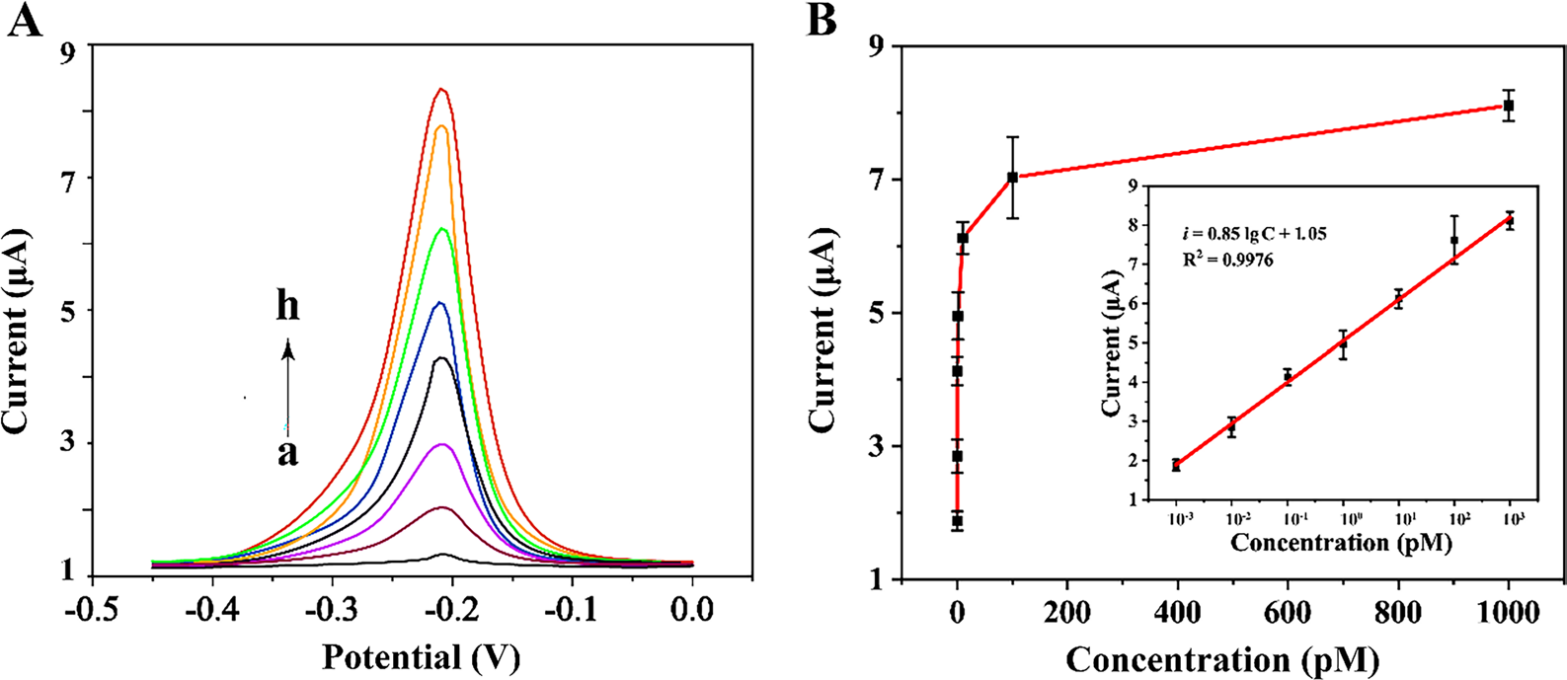
Evaluation of the sensitivity of the biosensor. **A** DPV curves responding to different concentrations of the target m^6^A DNA: **a** 0 pM, **b** 0.001 pM, **c** 0.01 pM, **d** 0.1 pM, **e** 1.0 pM, **f** 10 pM, **g** 100 pM, **h** 1000 pM. **B** The linear relationship between DPV signal and the logarithm of target m^6^A DNA concentration. All results expressed as mean  ±  standard variation (n  = 3). *Unpaired t test and one-way ANOVA with Tukey pairwise comparison (p  < 0.001)

Under optimal experimental conditions, the sensitivity of the biosensor was analyzed in the presence of different concentrations of target m^6^A DNA ranging from 1 fM to 1 nM. The DPV signal increased with the concentration of target m^6^A DNA (Fig. 4A), and the good linear relationship between the DPV response and the logarithm of target m^6^A DNA concentration ranging from 1 fM to 1 nM was obtained (Fig. 4B). According to the mathematical results, the determined linear regression equation was *i*  = 0.85 lg C  +  1.05 (correlation coefficient R^2^  =  0.9976, where *i* and C represents DPV signal and the concentration of target m^6^A DNA, respectively). The low detection of limit (LOD) for target m^6^A DNA was 0.89 fM according to the 3σ rule. The LOD was lower than most of previously reported sensing methods towards m^6^A DNA [14, 19, 20, 23], benefiting from the powerful signal amplification capability of MB@Zr-MOF. Moreover, the biosensor was time-saving compared to traditional strategies, the detailed discussion was depicted in Additional file 1: Table S3.

### Selectivity and stability of the biosensor

**Fig. 5:**
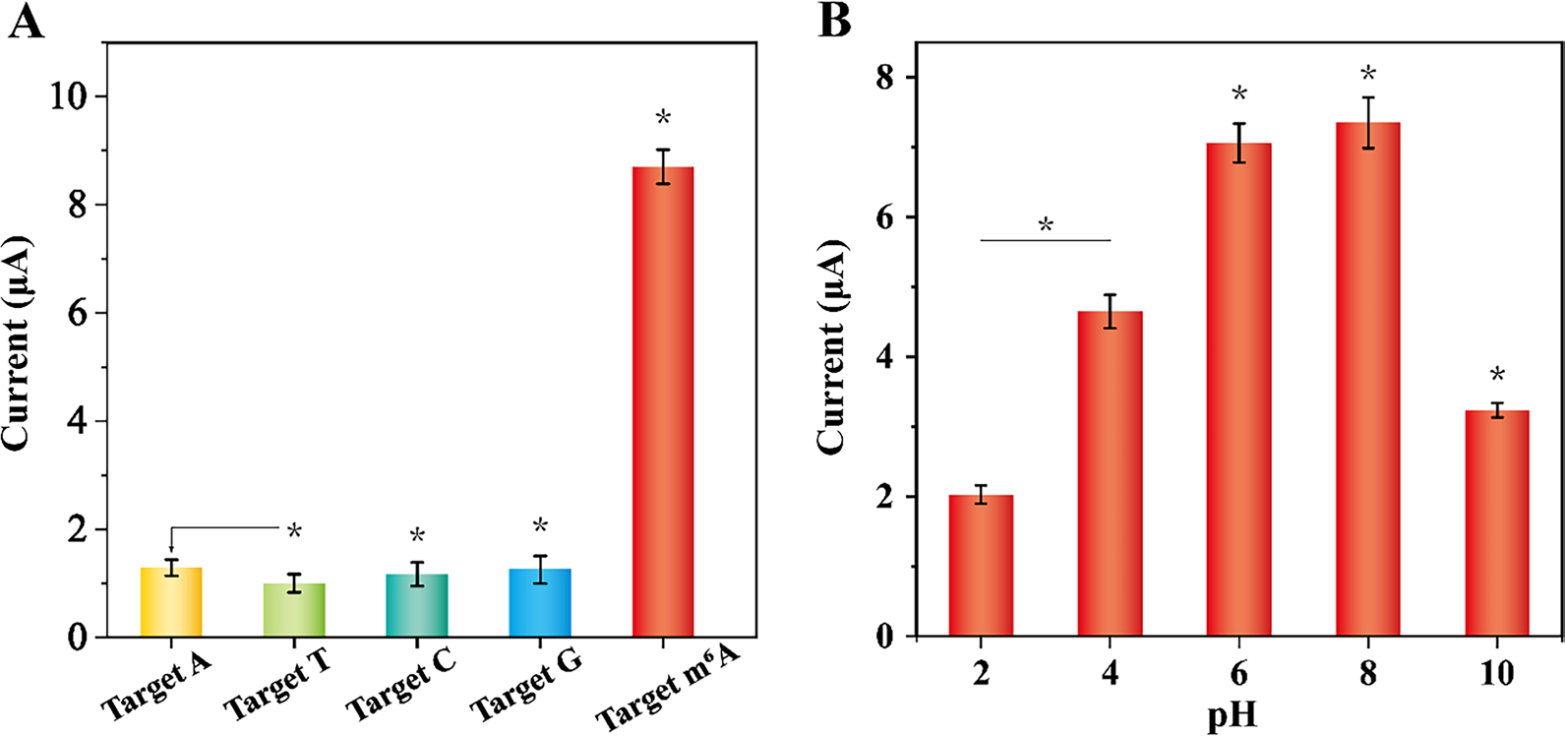
**A** Assessment of the selectivity of the developed biosensor. The concentrations of all DNA sequences were 1 nM. **B** Investigation of the stability of the developed biosensor at different pH. All results expressed as mean  ±  standard variation (n  = 3). *Unpaired t test and one-way ANOVA with Tukey pairwise comparison (p  < 0.001)

The selectivity of the proposed strategy was evaluated by replacing A site with normal T, C, and G bases, which were corresponding to target T, target C, and target G, respectively. As shown in Fig. 5A, the DPV signals of these interfering sequences were substantially identical to that of target A, and negligible change of electrochemical signal was observed. Nevertheless, the DPV signal of target m^6^A DNA had a significant increase, which was about 4.5-fold higher than that of these interfering sequences. These results demonstrated that the strategy exhibited high fidelity in discriminating target m^6^A DNA and other unmodified genomic DNA, which was attributed to the good selectivity of double-hindered replication towards target m^6^A DNA.

Furthermore, the stability of the developed biosensor was evaluated. Owing to acidic and alkaline condition may destroy the structure of LP@MB@Zr-MOF and disturb the loading of MB, pH could be a key parameter for the stability of the biosensor. As shown in Fig. 5B, the LP@MB@Zr-MOF could maintain stabilization with pH values changing from 6 to 8, and the highest DPV signal was obtained when the pH was about 8. In addition, the peak current values almost kept unchanged though the components of the developed biosensor were stored at 4 °C for 15 days (Additional file 1: Fig. S4). These results demonstrated that the good stability of the develop biosensor.

### Clinical applicability of the biosensor

To assess the applicability of the electrochemical biosensor towards detection of target m^6^A DNA in physiological environment, the recovery test was firstly carried out. In standard addition experiments, the comparable results of different concentrations of target m^6^A DNA at 10 fM, 1 pM, 100 pM in 20-fold diluted serum was illustrated in Additional file 1: Table S2. The recoveries rate was in the range of 96.65–103.02%, and the RSD was in the range of 4.34–5.87%. Furthermore, in order to test the applicability of this biosensor in real cells, the m^6^A DNA expressed in HepG2 cells and NeHepLxHT cells was analyzed by our developed method and ELISA kit [11]. As shown in Additional file 1: Fig. S5, with the increasing of concentration of HepG2 cells (a–c), the corresponding DPV signals increased proportionably, suggesting that the produced signal was highly dependent on the concentration of m^6^A DNA in HepG2 cells. And the signal of HepG2 cells (c) was obviously higher than that of NeHepLxHT cells (d) at the same concentration, which was matched with the previous reports [37]. Furthermore, the results of the electrochemical biosensor were consistent with that of ELISA kit. What’s more, compared with the commercial ELISA kit whose price is about RMB 6000–7000 for 48 assays [38], the proposed biosensor is much cheaper. These results proved that the proposed biosensor exhibited reliable capacity for sensing m^6^A DNA in cells and possessed great potential in clinical application.

## Conclusions

In summary, an ultrasensitive electrochemical biosensor for locus-specific detection of m^6^A DNA was developed based on double-hindered replication with nucleic acid-functioned MB@Zr-MOF. Integrating the impediment of m^6^A to DNA synthesis and the termination of m^6^A–Ag^I^–C mismatch’s instability to DNA replication, the biosensing strategy exhibited excellent performance in distinguishing between A site and m^6^A site. Utilizing MB@Zr-MOF as amplified signal indicator, the sensitivity of the biosensor was significantly improved, displaying LOD as low as femtomole level for analyzing m^6^A DNA. The developed method can be extended to detect m^6^A RNA in principle. Given the high sensitivity and excellent specificity, the electrochemical biosensor is a facile and alternative platform for the m^6^A DNA detection and biomedical researches. Despite these achievements, this developed strategy needs further optimization. For example, the relatively complicated synthesis of nucleic acid-functioned MB@Zr-MOF may limit its applications.

## Supplementary Information

**Additional file 1: Table S1.** DNA Sequences used in the experiment for established electrochemical biosensor. **Table S2. **The recoveries determined using the strategy by spiking m^6^A DNA into human serum samples. **Table S3.** The comparison of this method with other reports. **Figure S1.** Characterization of the modification procedure on an electrode. (A) CV and (B) EIS at (a) the bare GE, (b) capture probes immobilized electrode, (c) after blocked with MCH, (d) after hybridized with m^6^A DNA, (e) after hybridized with LP@MB@MOF. **Figure S2.** Optimization of the concentration of Zr-MOF. All results expressed as mean ± standard variation (n = 3). **Figure S3.** Optimization of the concentration of AgNO_3_. All results expressed as mean ± standard variation (n = 3). **Figure S4.** Evaluation of the stability of the developed biosensor. All results are expressed as mean ± standard variation (n = 3). **Figure S5.** Evaluation of the applicability of the developed biosensor in real cells. DNA extraction from 1 × 10^6^ HepG2 cells with (a)10-times dilution, (b) 5-times dilution, and (c) original value, and (d) DNA extraction from 1 × 10^6^ NeHepLxHT cells. All results are expressed as mean ± standard variation (n = 3).

## Data Availability

All data generated and analyzed during this study are included in this published article and additional file. The additional file is available. DNA Sequences used in the experiment for established electrochemical biosensor (Additional file 1: Table S1). The recoveries determined using the strategy by spiking m^6^A DNA into human serum samples (Additional file 1: Table S2). The comparison of this method with other reports (Additional file 1: Table S3). Characterization of the modification procedure on an electrode (Additional file 1: Figure S1). Optimization of the concentration of Zr-MOF and AgNO_3_ (Additional file 1: Figures S2, S3). Evaluation of the stability of the developed biosensor (Additional file 1: Figure S4). Evaluation of the applicability of the developed biosensor (Additional file 1: Figure S5).

## Abbreviations

m^6^A: N^6^-methyladenine
MB: Methylene blue
MOFs: Metal–organic frameworks
HCR: Hybridization chain reaction
RCA: Rolling circle amplification
CP: Capture probes
KF exo^−^: Klenow Fragment DNA polymerase (3′ → 5′ exo^−^)
LP: Linker probes
PBS: Phosphate buffer saline
MCH: Mercapto hexanol
HepG2: Hcuman hepatoellular carcinomas
CV: Cyclic voltammetry
EIS: Electrochemical impedance spectroscopy
DPV: Differential pulse voltammetry
PAGE: Polyacrylamide gel electrophoresis
DMEM: Dulbecco's modified eagle medium
TEM: Transmission electron microscopy
FT-IR: Fourier transform infrared spectra
LOD: Limit of detection
