# Supplementary material for: Femtomolar and locus-specific detection of N6-methyladenine in DNA by integrating double-hindered replication and nucleic acid-functionalized MB@Zr-MOF
Source: J Nanobiotechnology. 2021 Dec 7;19:408. doi: 10.1186/s12951-021-01156-0 (PMC8650346; doi:10.1186/s12951-021-01156-0)
Supplement: Supplementary file 1 — Additional file 1: Table S1. DNA Sequences used in the experiment for established electrochemical biosensor. Table S2. The recoveries determined using the strategy by spiking m6A DNA into human serum samples. Table S3. The comparison of this method with other reports. Figure S1. Characterization of the modification procedure on an electrode. (A) CV and (B) EIS at (a) the bare GE, (b) capture probes immobilized electrode, (c) after blocked with MCH, (d) after hybridized with m6A DNA, (e) after hybridized with LP@MB@MOF. Figure S2. Optimization of the concentration of Zr-MOF. All results expressed as mean ± standard variation (n = 3). Figure S3. Optimization of the concentration of AgNO3. All results expressed as mean ± standard variation (n = 3). Figure S4. Evaluation of the stability of the developed biosensor. All results are expressed as mean ± standard variation (n = 3). Figure S5. Evaluation of the applicability of the developed biosensor in real cells. DNA extraction from 1 × 10^6^ HepG2 cells with (a)10-times dilution, (b) 5-times dilution, and (c) original value, and (d) DNA extraction from 1 × 10^6^ NeHepLxHT cells. All results are expressed as mean ± standard variation (n = 3). [file 12951_2021_1156_MOESM1_ESM.docx]

**Supporting information**

**Femtomolar and Locus-Specific Detection of N6-Methyladenine in DNA by Integrating Double-hindered Replication and Nucleic Acid-functionalized MB@Zr-MOF**

*Qingyuan Zheng^a,1^, Tong Wang^a,1^, Xinmin Li^a,b^, Husun Qian^a^, Xintong Bian^a^, Xingrong Li^a^, Huijie Bai^a^, Shijia Ding^a^ and Yurong Yan^a,*^*

^a^ Key Laboratory of Clinical Laboratory Diagnostics (Ministry of Education), College of Laboratory Medicine, Chongqing Medical University, Chongqing 400016, China

^b^ Department of Laboratory Medicine, Chongqing Hospital of Traditional Chinese Medicine, Chongqing 400016, China

**Corresponding Authors**

*E-mail address: yanyurong@cqmu.edu.cn (Y. Yan).

**Author Contributions**

^1^ These authors contributed equally to this work.

**Table S1.** DNA Sequences used in the experiment for established electrochemical biosensor.

| **Name ^a^** | **Sequences (5’ to 3’)** |
| --- | --- |
| Prime | TCC TGC TTG TGG CAG |
| CP | AAAAAA TGG TGC TTG TGG CAG |
| LP | CGG AGC AAG GAG CGA AAAAAA |
| Target A | TCG CTC CTT GCT CCG A CTG CCA CAA GCA CCA |
| Target T | TCG CTC CTT GCT CCG T CTG CCA CAA GCA CCA |
| Target C | TCG CTC CTT GCT CCG C CTG CCA CAA GCA CCA |
| Target G | TCG CTC CTT GCT CCG G CTG CCA CAA GCA CCA |
| Target m^6^A | TCG CTC CTT GCT CCG m^6^A CTG CCA CAA GCA CCA |

**Table S2.** The recoveries determined using the strategy by spiking m^6^A DNA into human serum samples.

| **Target m^6^A**  **added (pM)** | **Detected**  **results (μA)** | **Recovery (%)** | **RSD (%) ^a^** |
| --- | --- | --- | --- |
| 0.01 | 2.85 ± 0.24 | 96.65 | 5.87 |
| 1.00 | 4.95 ± 0.32 | 102.62 | 4.70 |
| 100.00 | 7.03 ± 0.22 | 103.02 | 4.34 |
| **^a^** RSD, relative standard deviation. | | | |

**Table S3.** The comparison of this method with other reports.

| **Method** | **Detection time** | **Site-specific potential** | **Anneal process** | **Reference** |
| --- | --- | --- | --- | --- |
| ELISA | ~4 h | NO | NO | [1] |
| Mass spectrometry | ~4 h | NO | YES | [2] |
| Sequencing | ~5 h | NO | YES | [3] |
| PEL | ~3 h | NO | NO | [4] |
| Electrochemistry | ~2 h | YES | NO | This method |
| PEL: Photoelectrochemical immunosensor. | | | | |


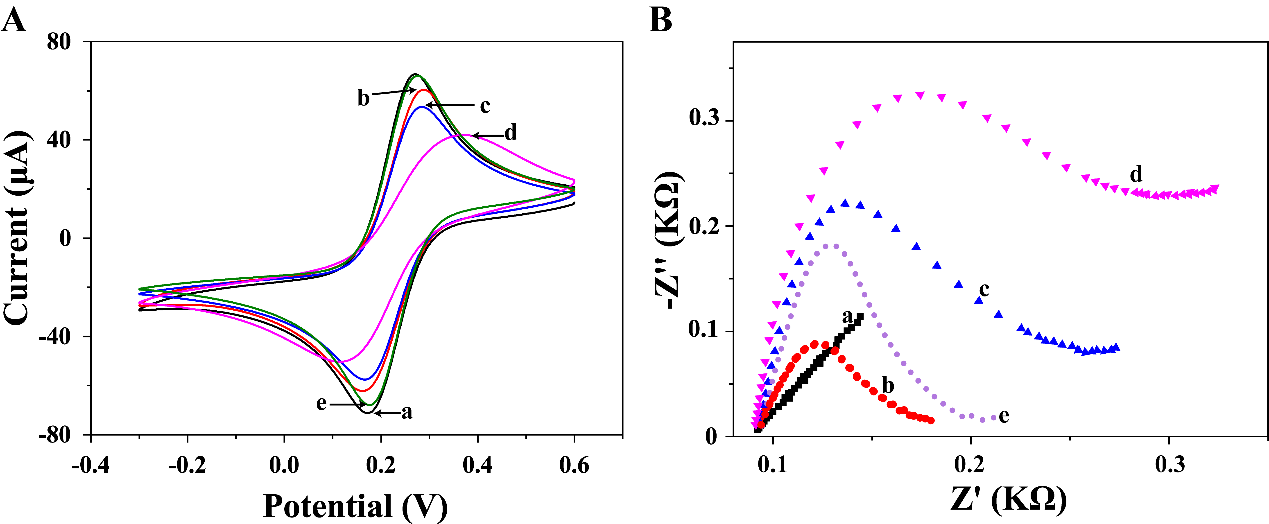


**Figure S1.** Characterization of the modification procedure on an electrode. (A) CV and (B) EIS at (a) the bare GE, (b) capture probes immobilized electrode, (c) after blocked with MCH, (d) after hybridized with m^6^A DNA, (e) after hybridized with LP@MB@MOF.


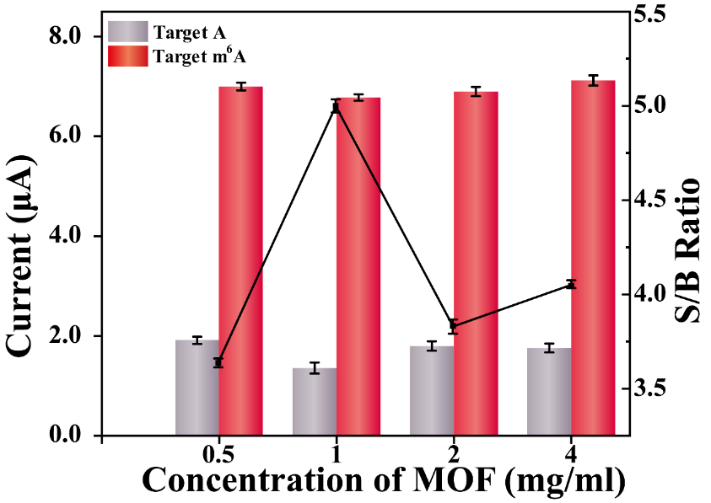


**Figure S2.** Optimization of the concentration of Zr-MOF. All results expressed as mean ± standard variation (n = 3).


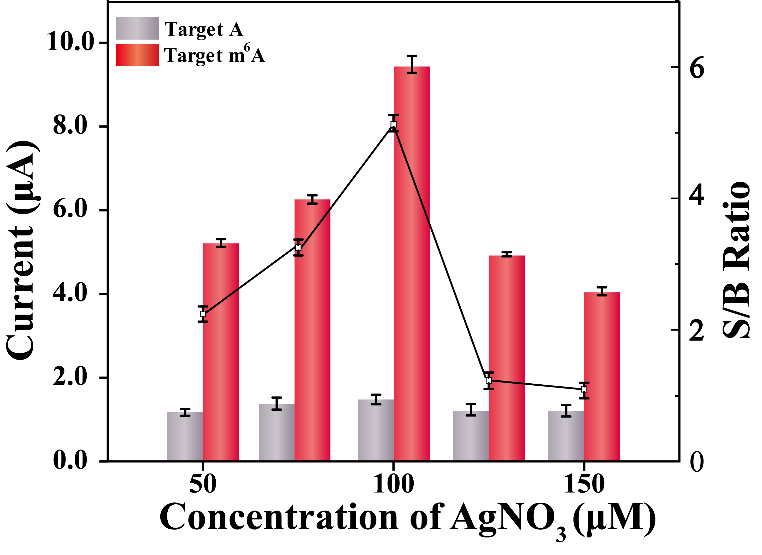


**Figure S3.** Optimization of the concentration of AgNO_3_. All results expressed as mean ± standard variation (n = 3).


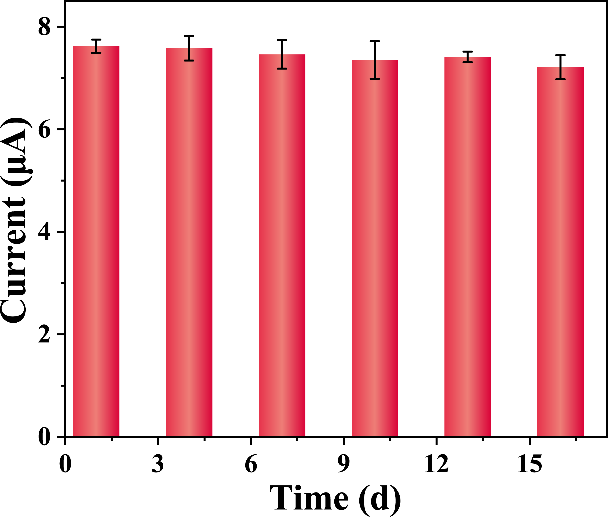


**Figure S4**. Evaluation of the stability of the developed biosensor. All results are expressed as mean ± standard variation (n = 3).


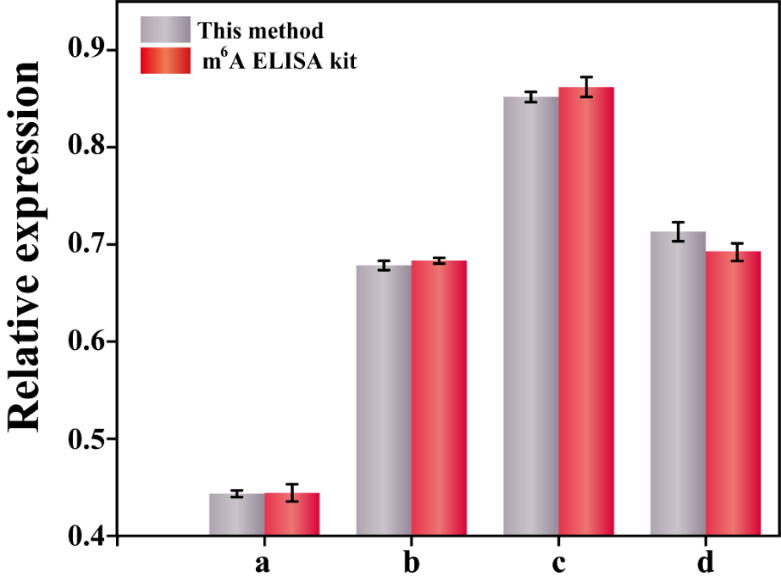


**Figure S5**. Evaluation of the applicability of the developed biosensor in real cells. DNA extraction from 1 × 10^6^ HepG2 cells with (a)10-times dilution, (b) 5-times dilution, and (c) original value, and (d) DNA extraction from 1 × 10^6^ NeHepLxHT cells. All results are expressed as mean ± standard variation (n = 3).

**REFERENCE**

1. Han Z, Wang X, Xu Z, Cao Y, Gong R, Yu Y, Yu Y, Guo X, Liu S, Yu M, et al. ALKBH5 regulates cardiomyocyte proliferation and heart regeneration by demethylating the mRNA of YTHDF1. Theranostics.2021; 11:3000-3016.

2. Boulias K, Greer E. Detection of DNA Methylation in Genomic DNA by UHPLC-MS/MS. Methods in molecular biology (Clifton, NJ).2021; 2198:79-90.

3. Flusberg BA, Webster DR, Lee JH, Travers KJ, Olivares EC, Clark TA, Korlach J, Turner SW. Direct detection of DNA methylation during single-molecule, real-time sequencing. Nat Methods.2010; 7:461-465.

4. Summerer D. N6-Methyladenine: A Potential Epigenetic Mark in Eukaryotic Genomes. Angewandte Chemie International Edition.2015; 54:10714-10716.s
